# Characterization of Scattered X-Ray Photons in Dental Cone-Beam Computed Tomography

**DOI:** 10.1371/journal.pone.0149904

**Published:** 2016-03-07

**Authors:** Ching-Ching Yang

**Affiliations:** Department of Medical Imaging and Radiological Sciences, Tzu-Chi University of Science and Technology, Hualien, Taiwan; Chongqing University, CHINA

## Abstract

**Purpose:**

Scatter is a very important artifact causing factor in dental cone-beam CT (CBCT), which has a major influence on the detectability of details within images. This work aimed to improve the image quality of dental CBCT through scatter correction.

**Methods:**

Scatter was estimated in the projection domain from the low frequency component of the difference between the raw CBCT projection and the projection obtained by extrapolating the model fitted to the raw projections acquired with 2 different sizes of axial field-of-view (FOV). The function for curve fitting was optimized by using Monte Carlo simulation. To validate the proposed method, an anthropomorphic phantom and a water-filled cylindrical phantom with rod inserts simulating different tissue materials were scanned using 120 kVp, 5 mA and 9-second scanning time covering an axial FOV of 4 cm and 13 cm. The detectability of the CT image was evaluated by calculating the contrast-to-noise ratio (CNR).

**Results:**

Beam hardening and cupping artifacts were observed in CBCT images without scatter correction, especially in those acquired with 13 cm FOV. These artifacts were reduced in CBCT images corrected by the proposed method, demonstrating its efficacy on scatter correction. After scatter correction, the image quality of CBCT was improved in terms of target detectability which was quantified as the CNR for rod inserts in the cylindrical phantom.

**Conclusions:**

Hopefully the calculations performed in this work can provide a route to reach a high level of diagnostic image quality for CBCT imaging used in oral and maxillofacial structures whilst ensuring patient dose as low as reasonably achievable, which may ultimately make CBCT scan a reliable and safe tool in clinical practice.

## Introduction

Cross-sectional radiography and computed tomography methods with multiplanar cross sections and 3D reconstructions are important diagnostic means in current dental and craniomaxillofacial medicine. Hard tissue visualization has been possible with conventional multi-detector CT (MDCT) for a long time, but there is the disadvantage of high radiation dose. Currently, the radiation dose associated with a typical MDCT scan (1–14 mSv) is comparable to the annual dose received from natural sources of radiation, such as radon and cosmic radiation (1–10 mSv) [1–3]. Dental cone-beam CT (CBCT) is an alternative to a MDCT scan that is appropriate for a wide range for craniomaxillofacial indications [4–6]. The radiation dose required is lower compared with conventional MDCT in most indications. In spite of the increasing use of CBCT to image the maxillofacial region, the relatively poor image quality of CBCT and particularly significant variability in CT numbers poses problems for its use as an effective tool in dentistry. To the contrary, MDCT produces relatively good image equality and stable CT numbers, mainly due to smaller inherent scatter signals as well as more linear detectors and sophisticated correction algorithms that have been developed over the past several decades. Scatter is a very important artifact causing factor in CBCT. The scatter-to-primary ratios (SPRs) are about 0.01 for single-ray CT and 0.05 to 0.15 for fan-beam and spiral CT, and may be as large as 0.4 to 2.0 in CBCT [7, 8]. Increased scatter not only amplifies patient dose but is a principal contributor to reduced contrast resolution and increased noise in CBCT images. Streak and cupping artifacts can also be produced, further degrading image quality [9, 10]. This work aimed to improve the image quality of dental CBCT through scatter correction. To reach this goal, a Monte Carlo simulator was built to characterize the scattered x-ray photons in dental CBCT. According to the research findings from the simulation results, a scatter correction strategy was proposed and validated using experimental measurements.

## Materials and Methods

### Dental cone-beam CT

CBCT scans were obtained from a dental CBCT scanner (3D eXam; KaVo Dental GmbH, Biberach, Germany), which uses an amorphous silicon flat panel detector of 23.8 cm width and 19.2 cm height. The nominal source-axis distance (SAD) is 49.54 cm and source-detector distance (SDD) is 71.4 cm. The x-ray tube operates at 120 kVp with a rotating 15 degree tungsten target and a focal spot of 0.5 mm. The nominal inherent filtration is 10 mm of aluminum equivalent thickness. A lead collimator is located adjacent to the x-ray beam window and can be used to adjust the cone-beam angle. The minimum and maximum axial field-of-view (FOV) were 4 cm and 13 cm, respectively, while the transverse FOV was fixed at 16 cm in diameter if the dental CBCT was acquired with full-fan mode, generating 301 projections within 360 degrees. A pixel matrix of 384x480 of 0.5 mm voxel size was obtained for one projection. The CBCT scans were reconstructed using the standard Feldkamp-Davis-Kress (FDK) algorithm [11] obtained from the OSCaR (Open Source Cone-beam Reconstructions) software package (available at http://www.cs.toronto.edu~/nrezvaniOSCaR.html) in a 536×536 matrix with a pixel size of 0.3 mm.

### Monte Carlo simulation

**Fig 1:**
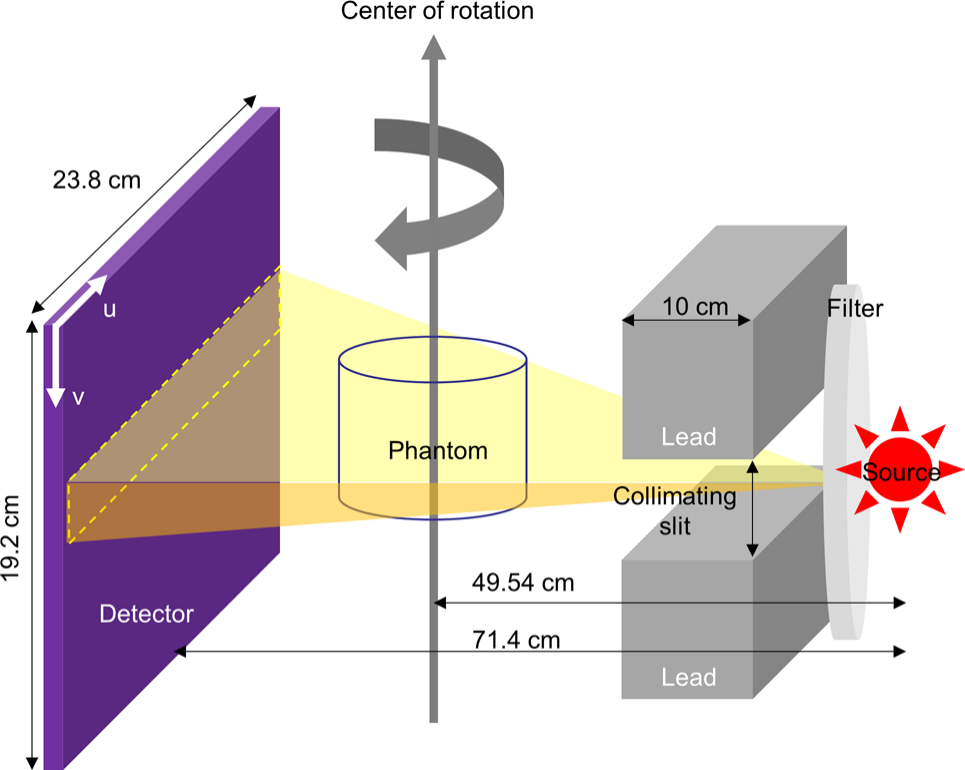
Illustration of the geometry of dental CBCT modeled with Monte Carlo simulation.

**Fig 2:**
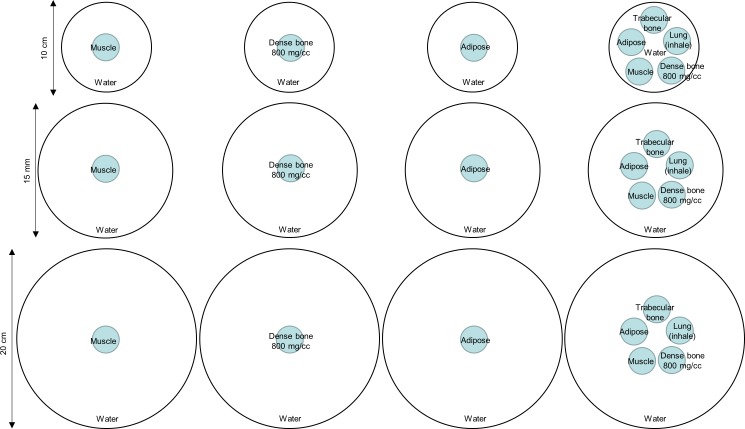
Illustration of the phantom settings in axial view used in Monte Carlo simulation.

The GEANT4 Application for Tomographic Emission (GATE) [12] version 6.0.0 was used to model the dental CBCT scanner (Fig 1), performed on a Linux cluster computer which had 8 quad core CPU slave nodes. CBCT acquisitions of water-filled cylindrical phantoms that were 10, 15 and 20 cm in diameter, 10 cm in height and had 3-cm-diameter rod inserts simulating muscle, dense bone at 800 mg/cc density, adipose, trabecular bone and lung (inhale) were simulated (Fig 2). For each phantom setting, CBCT scans were simulated with collimating slit of 0.5, 1, 2, 5, 10, 20 mm and without collimation. Hence, a total of 84 simulated CBCT scans has been conducted. In the GATE simulation of x-ray photons, every interaction process including the Compton and Rayleigh scatterings was labeled and the number of times that a photon undergoes the Compton or Rayleigh scatterings within the phantom or detector was counted [12, 13]. The output of the GATE simulation was in the list-mode ASCII format recording the coordinates, energy, time and scattering labels of the detected photons. The detected photons were separated into primary or scattered radiations first and then reformatted to form projection data. To characterize the scattered radiation in dental CBCT, the SPR defined as the ratio of scattered to primary radiation intensities was used to quantify the presence of scattered radiation in image signal. A central region-of-interest (ROI) of 11x11 pixels was placed on the total, primary and scatter projections to calculate the mean and standard deviation of photon intensity.

### Estimation of scattered x-ray photons

**Fig 3:**
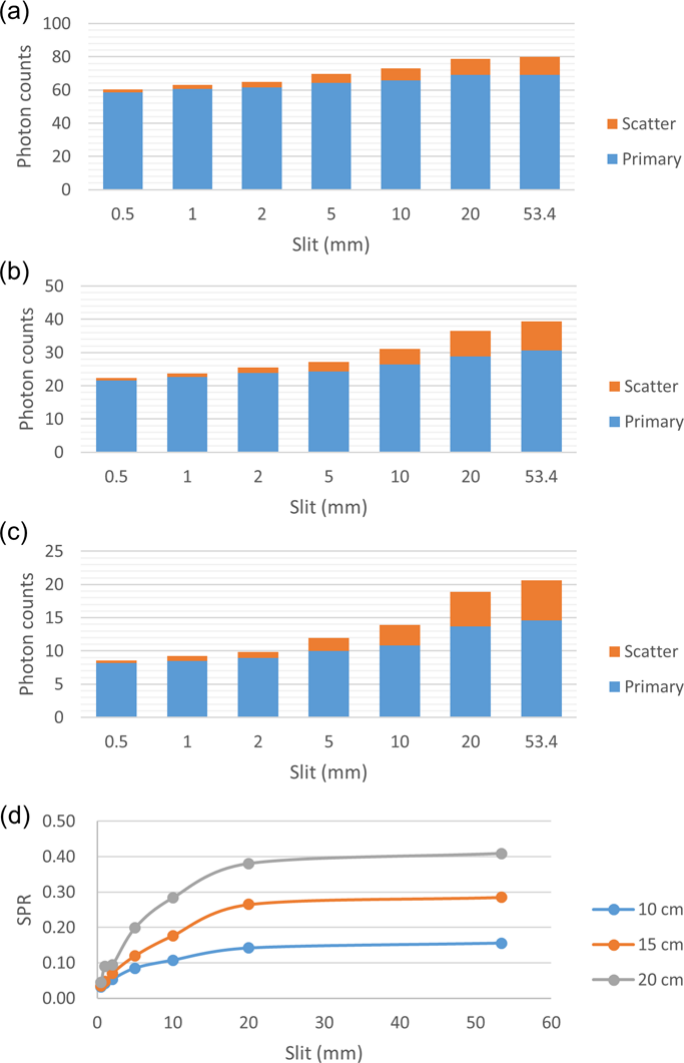
Primary and scattered radiation for (a) 10-cm-, (b) 15-cm-, and (c) 20-cm-diameter phantoms under various sizes of collimating slit. (d) Dependence of SPR on the size of collimating slit.

**Fig 4:**
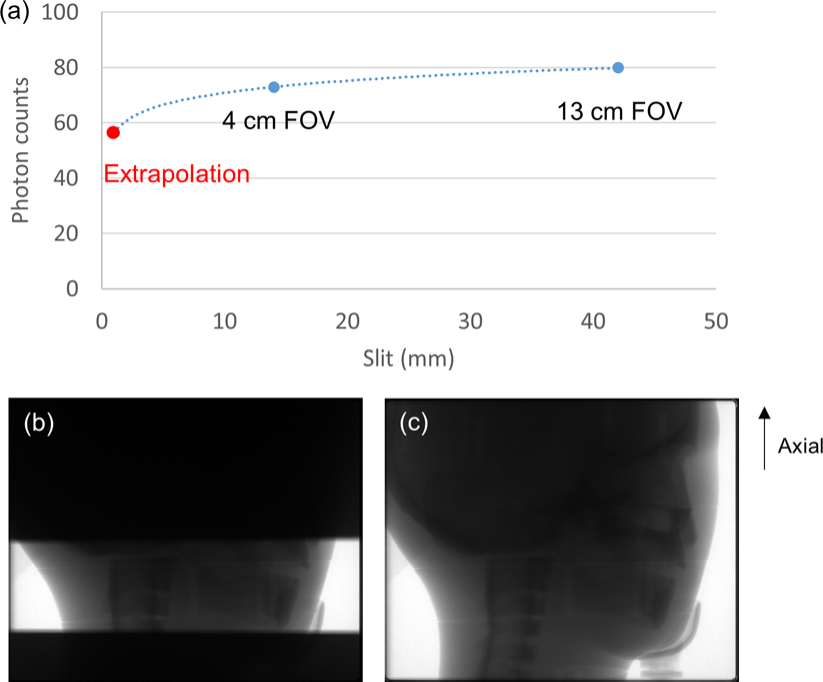
(a) An example of model fit to the projection data obtained from CBCT scans with (b) 4 cm axial FOV and (c) 13 cm axial FOV.

**Fig 5:**
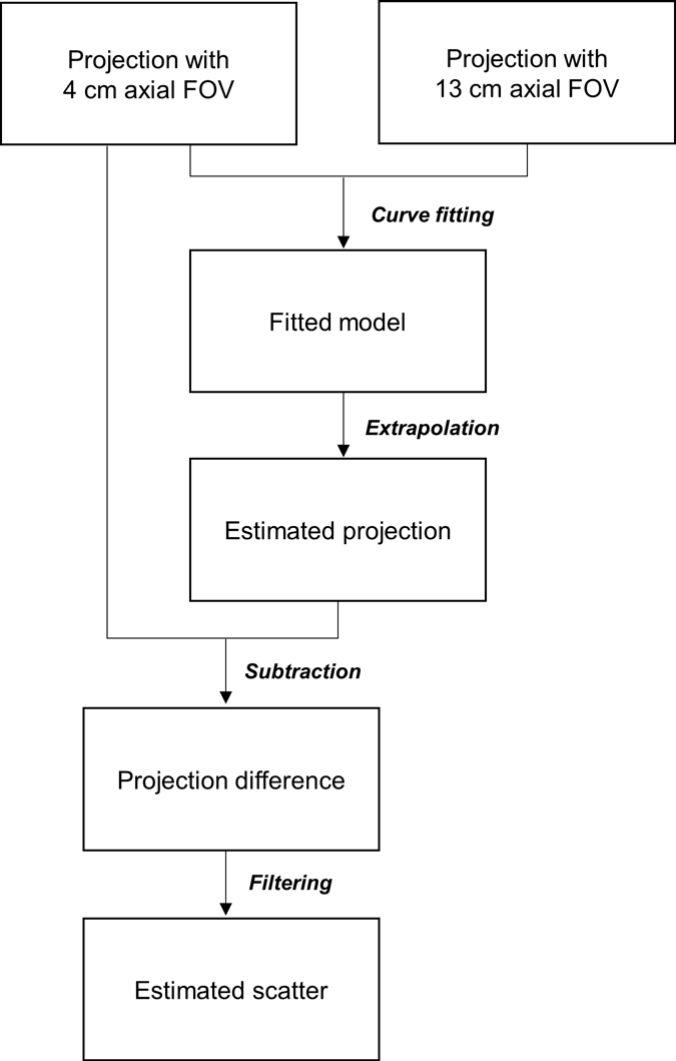
Flowchart of the scatter estimation scheme.

Collimation of the x-ray beam reduces the SPR by decreasing the contribution of peripheral scatter to the SPR in the FOV (Fig 3). Hence, a small axial FOV can be used to improve contrast-to-noise ratio (CNR) and reduce image artifacts. Since the SPR decreases as the beam size is reduced, it may be feasible to estimate a projection with negligible scatter contamination by extrapolating the model fitted to the projection data from CBCT scans with 2 different sizes of axial FOV (Fig 4). Projections obtained from CBCT scans with 4 cm and 13 cm axial FOV were used for curve fitting because they were the minimum and maximum sizes available on the CBCT scanner employed in this study, respectively. The projection estimated based on extrapolating the fitted model was quite noisy due to statistical fluctuations. As scatter signals have a dominant low-frequency component, the scatter projection was estimated from the low frequency component of the difference between the raw CBCT projection and the projection obtained by extrapolating the fitted model [14, 15]. A 2D Gaussian filter, with a window size of 11-by-11 pixels and a standard deviation of 5 pixels was applied to reduce the Poisson noise without affecting the low-frequency scatter signals. Fig 5 showed the flowchart of the scatter estimation scheme proposed in this work. The function for curve fitting was optimized by using Monte Carlo simulation. The coefficient of determination (R^2^) was calculated to assess the strength of the functional regression model. The estimated scatter projection was subtracted from the raw CBCT projection and was then reconstructed by using the standard FDK algorithm. In order to demonstrate the efficacy of the proposed scatter correction method, no other additional filter was applied on the projection data before FDK reconstruction.

### Experimental evaluation

**Fig 6:**
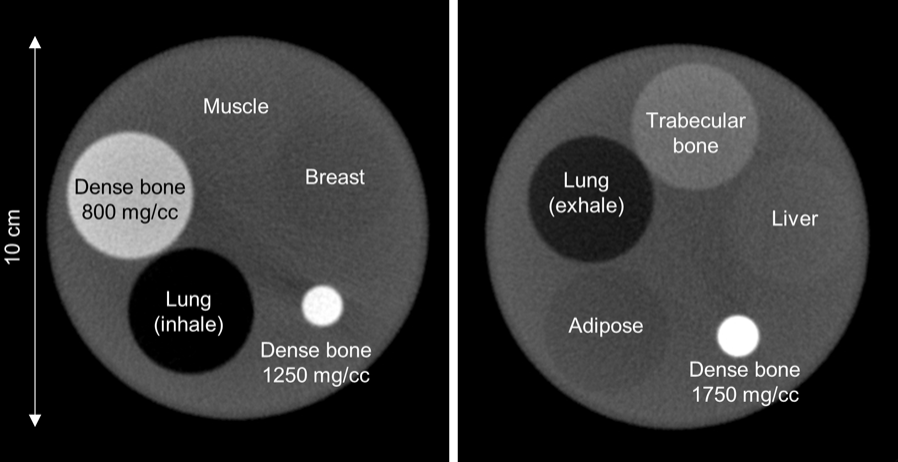
Axial CBCT images of the cylindrical phantom with two different phantom settings.

An anthropomorphic phantom (ATOM; CIRS, Norfolk, VA, USA) and a water-filled cylindrical phantom that was 10 cm in diameter, 10 cm in height and had 5 rod inserts simulating different tissue materials were scanned using 120 kVp, 5 mA and 9-second scanning time covering an axial FOV of 4 cm and 13 cm. Rod inserts from the electron density phantom (Model 062; CIRS, Norfolk, VA, USA) simulating lung (inhale and exhale), adipose, breast, muscle, liver, trabecular bone, dense bone at 800, 1250, 1750 mg/cc densities were inserted in the water-filled cylindrical phantom. Rod inserts are 3 cm in diameter and 5 cm in height, whereas the bone inserts have a core (1 cm in diameter, 5 cm in height) surrounded by soft tissue equivalent epoxy resin. Fig 6 showed the axial CBCT images acquired with 4 cm-axial-FOV scan of the cylindrical phantom with two different phantom settings. The Gafchromic XR-QA2 films (International Specialty Products, Wayne, NJ, USA) were used to measure the size of collimating slit and axial FOV of CBCT scans. For the CBCT data acquired with the anthropomorphic phantom, the mean and standard deviation within a ROI centered on the first view of the projection data and the central slice of the reconstructed images were calculated to evaluate the image noise introduced by different data acquisition and processing procedures. The central ROI consists of 3528 pixels in the projection domain and 10029 pixels in the image domain. For the CBCT data acquired with the water-filled cylindrical phantom, the detectability of CBCT images was evaluated by calculating the CNR defined as:

$$CNR=\frac{\left|CT\#-CT{\#}_{water}\right|}{SD_{water}}$$ (1)

where CT# is the mean CT number of rod inserts, CT#_water_ and SD_water_ are the mean and standard deviation of CT numbers of water-filled cylindrical phantom, respectively. A CNR of 1.0 occurs when the image contrast (or difference) between rod insert and background was equal to the background noise. A circular ROI with diameter of 0.9 cm was placed in the rod inserts and the water-filled cylindrical phantom to calculate the mean and standard deviation of CT numbers. The image processing tools were implemented in MATLAB 7.1 (The Mathworks, Natick, MA, USA).

## Results

**Fig 7:**
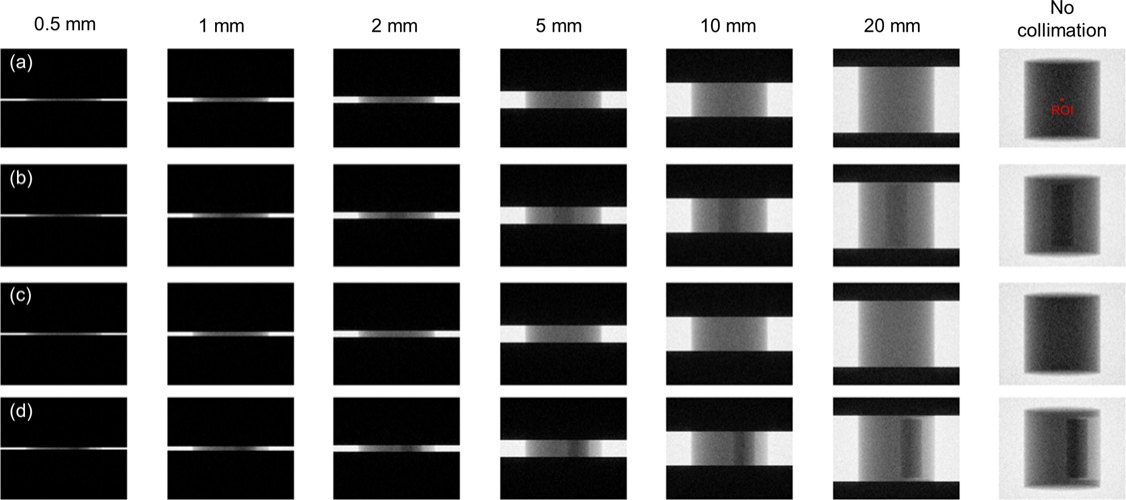
Simulated projections acquired with various sizes of collimating slit for the 10-cm-diameter phantom with rod inserts simulating (a) muscle, (b) bone, (c) adipose, (d) 5 different tissue types.

**Fig 8:**
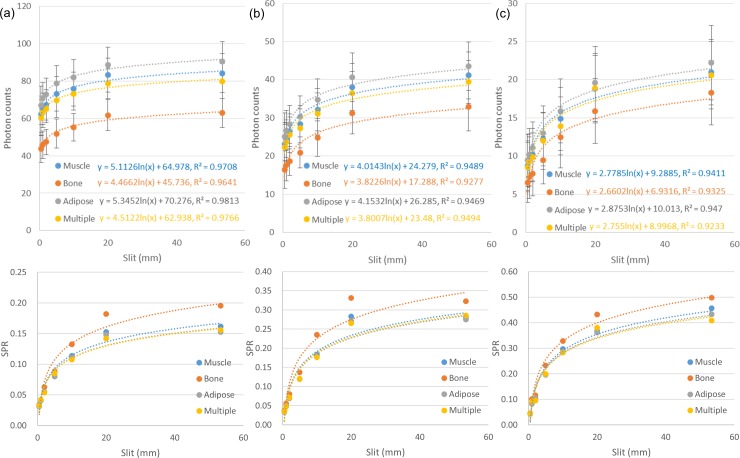
Photon intensity in simulated projections acquired with various sizes of collimating slit (top row) and the corresponding SPR (bottom row) for (a) 10-cm-, (b) 15-cm- and (c) 20-cm-diameter phantoms with rod inserts simulating muscle, bone, adipose and 5 different tissue materials.

**Fig 9:**
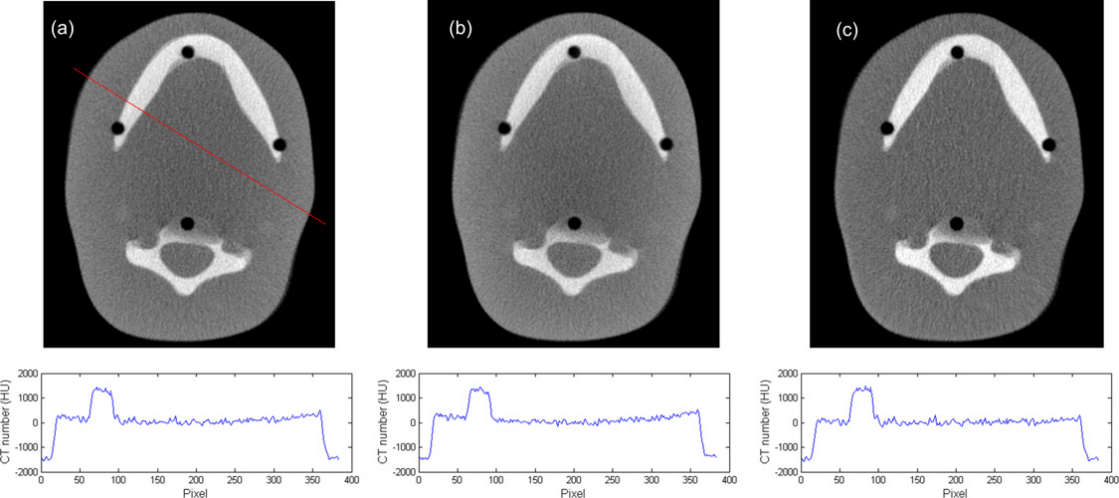
Axial CBCT images and intensity profiles through the red line of the anthropomorphic phantom acquired using (a) 4-cm-FOV scan, (b) 13-cm-FOV scan, and (c) 4-cm-FOV scan corrected by the proposed method.

**Fig 10:**
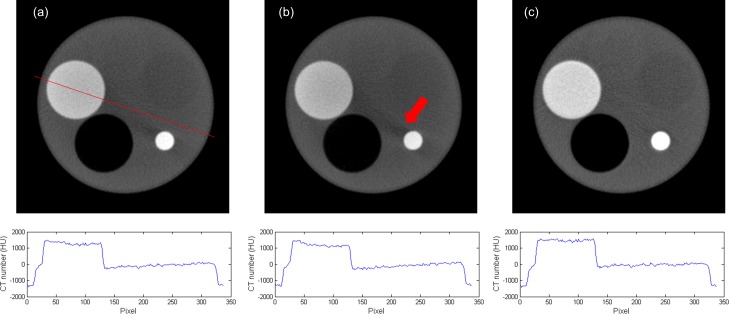
Axial CBCT images and intensity profiles through the red line of the cylindrical phantom with the first phantom setting acquired using (a) 4-cm-FOV scan, (b) 13-cm-FOV scan, and (c) 4-cm-FOV scan corrected by the proposed method.

**Fig 11:**
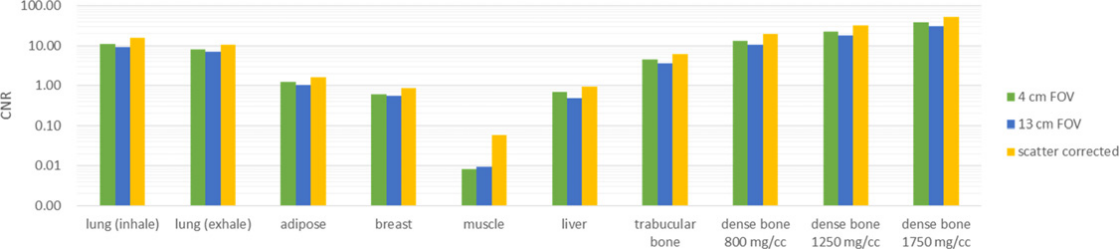
CNR of the rod inserts in the cylindrical phantoms from CBCT images acquired using 4-cm-FOV scan, 13-cm-FOV scan and 4-cm-FOV scan corrected by the proposed method.

Fig 7 demonstrated the simulated total projections (primary + scatter) acquired with various sizes of collimating slit for the 10-cm-diameter phantoms with rod inserts simulating various tissue materials, respectively. A rectangular ROI of 11x11 pixels used to calculate the mean amount of photon intensity was also shown. Fig 8 demonstrates the photon intensity in simulated total projections (primary + scatter) acquired with various sizes of collimating slit and the corresponding SPR for the 10-cm-, 15-cm- and 20-cm-diameter phantoms with rod inserts simulating muscle, bone, adipose and 5 different tissue materials. It can be seen that the total radiation and SPR decreased as the collimating slit was reduced. The x and y in the regression models shown in Fig 8 were the size of collimating slit and the mean value of total radiation within the ROI, respectively. The R^2^ of regression models ranged from 0.9641–0.9813, 0.9277–0.9489 and 0.9233–0.9470 for the 10-, 15- and 20-cm-diameter phantoms, respectively, indicating an excellent fit to the simulation data. Hence, the natural logarithmic function was used to fit the 4-cm-FOV and 13-cm-FOV projections obtained from experimental measurements on a pixel-by-pixel basis, i.e., each pixel in the projection data has its regression model. According to the results obtained using XR-QA2 films, the collimating slit was 14 mm and 42 mm for the 4-cm-FOV and 13-cm-FOV projections, respectively. Figs 9 and 10 show axial CBCT images and intensity profiles through the red line of the anthropomorphic phantom and the cylindrical phantoms. Beam hardening (red arrows) and cupping artifacts were observed in CBCT images without scatter correction, especially in those acquired with a 13 cm FOV. As seen in Figs 9C and 10C, these artifacts were reduced after performing the proposed scatter correction method. Table 1 lists the mean and standard deviation within the central ROI in the projection domain and the image domain for the CBCT data of the anthropomorphic phantom. The standard deviation for the CBCT data corrected by the proposed method was reduced compared with the projections estimated by extrapolating the fitted model, but was slightly higher than those from 4-cm-FOV and 13-cm-FOV scans. Fig 11 summarizes the CNR of the rod inserts in the cylindrical phantoms. The CNR from 13-cm-FOV scans was the lowest among the 3 groups. Compared with the CBCT images acquired with 4 cm FOV without scatter correction, CBCT images corrected with the proposed method demonstrated improved CNR. The average CNR over 10 different tissue types for CBCT images acquired using 4-cm-FOV scan, 13-cm-FOV scan and 4-cm-FOV scan corrected by the proposed method was 10.13, 8.17 and 14.19, respectively.

**Table 1:** Mean and standard deviation within the central ROI in the projection domain and the image domain.

	Projection (photon counts)		Reconstructed image (HU)	
	Mean	Standard deviation	Mean	Standard deviation
4 cm FOV ^a^	7095	739	4.51	87.61
13 cm FOV ^b^	8167	703	3.89	85.82
estimated ^c^	5497	866	9.13	284.62
scatter corrected ^d^	5491	785	5.90	98.76

a: projection data were obtained from 4-cm-FOV scan

b: projection data were obtained from 13-cm-FOV scan

c: projected data were estimated by extrapolating the fitted model

d: projection data were obtained by correcting 4-cm-FOV scan using the proposed method.

## Discussion

Conventional dental radiography is based on 2D representation of 3D structure and hence suffers from several geometric effects such as magnification, distortion and superimposition [16, 17]. The introduction of CBCT in dentistry heralds a true paradigm shift from a 2D to a 3D approach. Dental CBCT allows visualization of the complex relationships in oral and maxillofacial structures, and its use has increased rapidly over the last decade in various dental areas such as orthodontics, endodontics, periodontics, implantology, restorative dentistry, and dental and maxillofacial surgery [18, 19]. The CBCT devices designed for dental imaging are inherently different from the conventional MDCT scanners. In conventional fan-beam MDCT, collimation at the x-ray source restricts the axial coverage of the beam, only allowing scatter from a thin axial volume of tissue to reach the detector elements during section acquisition. In contrast, CBCT expands the axial coverage of the beam, allowing x-ray scatter generated from the entire volume of coverage to reach the detector elements as the image is acquired. As is common with CBCT systems, voxel values exhibit a systematic error primarily associated with high x-ray scatter [20]. In addition, increased scatter in CBCT imaging is a principal contributor to reduced contrast resolution in comparison with MDCT. Because of the divergence of the x-ray beam over the area detector in CBCT, there is resultant nonuniformity of the incident x-ray beam on patients, with greater noise on the cathode side relative to the anode side, i.e., the heel effect.

The contamination of CT data with scattered radiation depends on the geometry of the CT scanner and object under study. Numerous investigations have been undertaken to characterize scatter radiation in radiographic imaging. Among various strategies, Monte Carlo simulation can realistically reproduce actual acquisition and is widely accepted as the least biased method to investigate the interaction of photon in tissue. In this work, simulation results were used to investigate the relationship between the size of the collimating slit and the SPR, and it was found that the natural logarithmic function can be used to describe this relationship. Because Monte Carlo simulation still requires significant computation time, the fitted model for each pixel in the projection data was not determined using simulation only. Instead, the curve-fit parameters for the natural logarithmic function were determined by fitting to the 4-cm-FOV and 13-cm-FOV projections obtained from experimental measurements on a pixel-by pixel basis. Hence, the fitted models include information from Monte Carlo simulation (i.e., the natural logarithmic function) and experimental measurements (i.e., the curve-fit parameters of the fitted model). Due to the inclusion of experimental measurements, the characteristics of the geometry of the CT scanner and object under study were introduced into the fitted models, so the proposed method should be robust to patient orientation and asymmetry. In Figs 9 and 10, it was observed that the cupping artifacts were reduced in the CBCT images corrected by the proposed method, demonstrating its efficacy on scatter correction and its robustness to the orientation and asymmetry of object under study. The underestimation of attenuation profiles for the cone beam geometry is due to the contamination of projection data with scattered radiation, which reduces CT numbers in the central area of reconstructed images and thus creates cupping artifacts. Compared with a large axial coverage, a small axial FOV which reduces the contribution of scatter into the FOV is a possible approach to reduce the cupping artifacts. However, the cupping artifacts can still be observed in CBCT images acquired with 4-cm-FOV scan, which indicates that some sort of scatter correction algorithm should be used to further improve the image quality. Besides the cupping artifacts, the loss of target detectability is one of the most well-known phenomena caused by scattered radiation, so the CNR was employed to determine the detectability of object within reconstructed images. Although a slightly higher noise level was observed in CBCT data corrected by the proposed method when compared with the original CBCT data, improved target detectability quantified as CNR was found in the reconstructed images corrected by the proposed method. To further improve the image quality of CBCT data corrected by the proposed method, additional noise reduction filter can be applied on the projection data before FDK reconstruction.

Since the cancer risk associated with the radiation dose in CT is not zero, it is clear that reducing radiation dose in CT must continue to be one of the top priorities in medical imaging community. Several studies demonstrate that CBCT technologies seem to provide reduced doses as compared with conventional MDCT. Lodlow et al reported that the effective doses for CBCT scanner decreased with smaller FOV scans [6]. According to their results, the effective dose ranged from 68–1073 μSv for a large FOV scan (> 15 cm) and 69–506 μSv for a medium FOV scan (10–15 cm). At a comparable medium FOV, a 64-slice MDCT produced 530 μSv at default settings and 860 μSv using a dose-sparing protocol. Loubele et al reported that higher effective dose was observed in MDCT (2 scanners: 474–1160 μSv) than in CBCT (3 scanners: 13–82 μSv) [4]. Generally in CT, the larger the FOV, the higher the radiation exposure to patients. Pauwell et al reported a 20-fold range in effective dose of 14 CBCT scanners being examined (19–368 μSv) [21]. According to the results from Theodorakou et al [22], the effective doses of 4-cm-FOV and 13-cm-FOV CBCT scans performed in this work were about 63 μSv and 134 μSv for 10-year-old child, respectively, and were about 49 μSv and 82 μSv for adolescents, respectively. Hence, the effective dose of the proposed method could be around 197 μSv and 131 μSv for 10-year-old child and adolescents, respectively. The effective doses for adults are expected to be lower than those for adolescences due to the decreased radiosensitivity of adults compared to children. Despite the lower doses of CBCT compared with MDCT, strict adherence to selection criteria and imaging protocols to ensure patient radiation dose as low as reasonable achievable is recommended. In CBCT, exposure parameters such as tube voltage, tube current, exposure time and slice thickness can be optimized to reduce patient dose. Increase beam filtration has the potential to reduce patient dose by reducing the number of low-energy photons in the x-ray beam. Ludlow et al have reported an average exposure reduction of 43% when the CBCT scanner was equipped with additional filtration [5]. In addition to the hardware-based dose reduction strategies, patient positioning modifications (tilting the chin) and use of additional personal protection (thyroid collar) can substantially reduce the dose up to 40% [7].

Several limitations to this study need to be acknowledged. First, all images were acquired with the anthropomorphic phantom and cylindrical phantom with rod inserts simulating different tissue materials. Second, the reconstruction algorithm and data processing approaches can influence the study results. Third, the proposed scatter correction method was verified using experimental measurements obtained from only 1 CBCT scanner. Additional clinical studies assessing scattered radiation for different CBCT scanners will be needed and valuable. Hopefully the calculations performed in this work can provide a route to reach a high level of diagnostic image quality for CBCT imaging used in oral and maxillofacial structures whilst ensuring patient dose as low as reasonably achievable, which may ultimately make CBCT scan a reliable and safe tool in clinical practice.

## Conclusion

A Monte Carlo simulator has been built to characterize the scattered x-ray photon in dental CBCT. According to the research findings of the simulation results, a scatter correction method has been proposed and validated using experimental measurements. The beam hardening and cupping artifacts were reduced in the CBCT images corrected by the proposed method, which contributed to the improved target detectability quantified as CNR. Hopefully the calculations performed in this work can provide a route to reach a high level of diagnostic image quality for CBCT imaging used in oral and maxillofacial structures whilst ensuring patient dose as low as reasonably achievable, which may ultimately make CBCT scan a reliable and safe tool in clinical practice.
